# Cost-effectiveness of a proactive, integrated primary care approach for community-dwelling frail older persons

**DOI:** 10.1186/s12962-019-0181-8

**Published:** 2019-07-09

**Authors:** Lotte Vestjens, Jane M. Cramm, Erwin Birnie, Anna P. Nieboer

**Affiliations:** 1 Department of Socio-Medical Sciences, Erasmus School of Health Policy & Management, Erasmus University Rotterdam, P.O. Box 1738, 3000 DR Rotterdam, The Netherlands; 2 Department of Genetics, University Medical Center Groningen, University of Groningen, P.O. Box 30001, 9700 RB Groningen, The Netherlands

**Keywords:** Economic evaluation, Primary care, Integrated care, Frailty, Elderly, Quasi-experimental design

## Abstract

**Background:**

The article reports on the cost-effectiveness of the proactive, integrated primary care program *Finding and Follow*-*up of Frail older persons* (FFF) compared with usual primary care for community-dwelling frail older persons in the Netherlands.

**Methods:**

This study had a matched quasi-experimental design (pretest and posttest). The economic evaluation was performed from a healthcare perspective with a time horizon of 12 months. The target population consisted of community-dwelling frail older persons aged ≥ 75 years in the FFF intervention group (11 general practitioner (GP) practices) and in the control group receiving usual care (4 GP practices). The effectiveness measures for the cost-effectiveness and cost-utility analyses were subjective well-being (Social Production Function Instrument for the Level of well-being short; SPF-ILs) and QALYs (EuroQol; EQ-5D-3L), respectively. Costs were assessed using resource use questionnaires. Differences in mean effectiveness between groups were assessed using univariate, multilevel and propensity score matched analyses, with and without imputation of missing values. Differences in costs were assessed using Mann–Whitney *U*-tests and independent samples *t*-tests. Bootstrapping was performed, and predicted incremental cost-effectiveness ratios (ICERs) and incremental cost-utility ratios (ICURs) were depicted on cost-effectiveness planes.

**Results:**

The various analyses showed slightly different results with respect to differences in estimated costs and effects. Multilevel analyses showed a small but significant difference between the groups for well-being, in favor of the control group. No significant differences between groups in terms of QALYs were found. Imputed data showed that mean total costs were significantly higher in the intervention group at follow-up.

**Conclusion:**

Proactive, integrated care for community-dwelling frail older persons as provided in the FFF program is most likely not a cost-effective initiative, compared with usual primary care in the Netherlands, in terms of well-being and QALYs over a 12-month period.

**Electronic supplementary material:**

The online version of this article (10.1186/s12962-019-0181-8) contains supplementary material, which is available to authorized users.

## Background

This article reports on the cost-effectiveness of a proactive, integrated primary care approach compared with usual primary care for community-dwelling frail older persons in the Netherlands. We evaluated the *Finding and Follow*-*up of Frail older persons* (FFF) approach, which aims to maintain or improve older people’s well-being and is implemented by part of the Dutch general practitioners (GPs). The FFF approach consists of proactive identification of frail older persons in the community and subsequent multidisciplinary (including professionals with geriatric expertise) consultations and individualized follow-up coordinated by case managers. Integrated care and support is widely acknowledged to be a key initiative in improving care and support for older persons [1]. In addition, integrated care approaches, like the FFF program, may help to maintain community-dwelling frail older persons’ well-being [2]. Over the years, a shift has occurred from a disease-oriented care model toward a more proactive and integrated approach [3]. Traditional disease-specific care delivery approaches for frail older persons, who often have multiple conditions, do not meet these individuals’ comprehensive (healthcare) needs [4–8]. Moreover, frailty has been associated with increased utilization of primary, hospital, and nursing home care [9, 10]. The provision of high-quality care and support to the growing number of frail older persons poses a challenge [11, 12], and the comprehensive (healthcare) needs of this population place a burden on healthcare resources [13]. Integrated care initiatives are assumed to improve quality of care and ultimately aim to enhance patient outcomes while making efficient use of healthcare resources [14, 15]. Important elements of integrated care are: (i) a proactive approach that is coordinated effectively around a person’s health and social care needs; (ii) a patient-centered approach in which a person is involved in decision-making and care processes, and the person’s needs are taken into consideration; (iii) an approach in which multiple interventions are delivered (simultaneously); and (iv) a multidisciplinary approach in which professionals from multiple disciplines are involved [3]. GPs are considered to be key actors in the implementation of promising initiatives targeting frail older persons [9]. Many integrated care initiatives have emerged and are implemented in the primary healthcare sector, but evidence of their effectiveness and cost-effectiveness remains mixed [3, 16–21]. Integrated primary care programs for frail older persons have shown no effect on the majority of outcomes, and evidence for their cost-effectiveness is limited [16]. Although the FFF approach has been found to have positive effects on the quality of care as perceived by healthcare professionals [22], and to achieve improvements in older persons’ perceived care quality and coproduction of care over time [23], its cost-effectiveness has yet to be investigated. Therefore, the aim of the present study was to evaluate the cost-effectiveness of the FFF approach in a population of community-dwelling frail older persons.

Methods **Design, setting and participants**

**Fig. 1:**
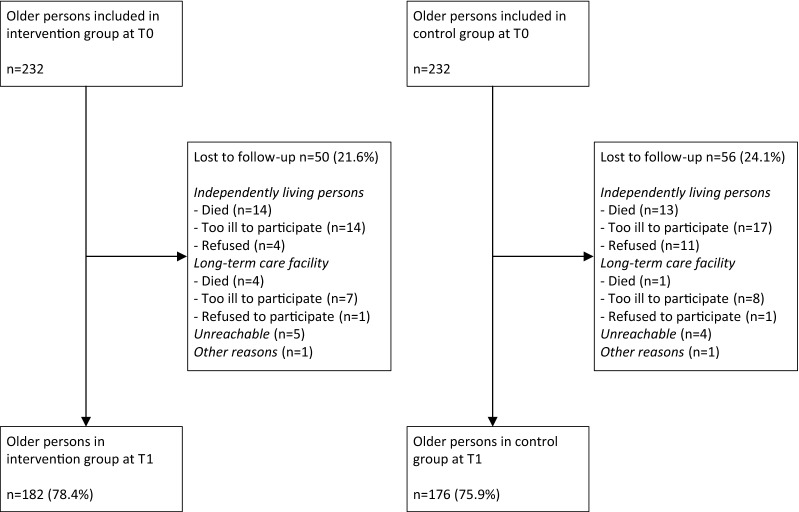
Flowchart of study participation

This longitudinal evaluation study had a matched quasi-experimental design with one pretest and one posttest (12-month follow-up period). The study was conducted in 15 GP practices located in the western part of North Brabant Province, the Netherlands, between 2014 and 2017. The intervention group consisted of community-dwelling frail persons aged 75 years and older who were registered at 11 GP practices that implemented the proactive, integrated primary care approach FFF. The control group consisted of community-dwelling frail older persons (≥ 75 years of age) who were registered at 4 GP practices that delivered usual primary care. Written informed consent to participate in the study was obtained from all participants. All participants in the intervention group were individually matched one-to-one to participants in the control group based on sex (male/female), educational level (low/high), and frailty score. As shown in Fig. 1, each group consisted of 232 frail older persons at baseline. At T1, 182 older persons remained in the intervention group and 176 older persons remained in the control group (loss to follow-up rates of 21.6% and 24.1% respectively). The medical research ethics committee of the Erasmus Medical Centre in Rotterdam, the Netherlands, concluded that the rules laid out in the Medical Research Involving Human Subjects Act did not apply (study protocol number MEC-2014-444). More details of the study design have been published elsewhere [24].

**Usual primary care**

Compared with the primary care systems in many countries in Europe, the Dutch primary care system is strongly developed. Many different (healthcare) providers, including GPs, primary care psychologists and physiotherapists, are involved in primary care delivery in the Netherlands [25]. GPs have a central role in the healthcare sector and a strong gatekeeping function [26], implying that referral is generally necessary to access most hospital and specialist care [25, 27]. Each patient is registered at a GP practice of his or her choice, usually located in the person’s neighborhood. GPs are commonly patients’ first contact with the healthcare system, and most first encounters take place after the occurrence of a (medical) problem. In general, GPs in the Netherlands are considered to be non-interventionist, resulting in relatively low prescription and referral rates. In comparison with GPs in other European countries, Dutch GPs provide a broad scope of (healthcare) services to their patients. Collaboration between GPs and (practice) nurses is common [25]. An example of a nurse-led service is the provision of diabetes management in primary care [27]. In the care for community-dwelling frail older persons, GPs can consult elderly care physicians with expertise in geriatric medicine [28]. The primary care system lacks, however, sufficient coordination and continuity (with specialist care) and is reactive and characterized by fragmentation [29]. Frail older persons in this study’s control group received usual care services provided by their GP practices and local health and community organizations. For a detailed description of care delivery and implemented interventions in control and intervention GP practices, see Vestjens, Cramm, and Nieboer [22].

Intervention Frail older persons in the intervention group received primary care according to the FFF approach. This approach combines several interrelated components (see Table 1) with the aim of providing high-quality proactive, integrated primary care for frail community-dwelling older persons. A decline of the well-being of community-dwelling frail older persons may be expected over time [2]. Consequently, the aim of the FFF program is to maintain or improve frail older people’s well-being and protect against its deterioration. The FFF approach is implemented in GP practices and led by GPs. Community-dwelling older patients registered at the GP practices are screened for frailty using the Tilburg Frailty Indicator (TFI) [30] during a home visit by the practice nurse, homecare nurse or geriatric nurse. This 15-item questionnaire assesses frailty in the physical, psychological, and social domains. Scores range from 0 to 15, and persons with scores ≥ 5 are identified as frail [30]. Persons with TFI scores < 5 can also be identified as frail based on additional examination by professionals. Problems and needs are reported in multiple domains according to the SFSPC-model, i.e., somatic (e.g., pain, fall risk), functional (e.g., limitations in activities of daily living like problems with eating or household activities), social (e.g., social network), psychological (e.g., fear, coping, depression), and communication (e.g., visual or hearing impairments). Outcomes of this in-home assessment are reported and discussed with the GPs and elderly care physicians, i.e., physicians in primary care with expertise in geriatric medicine [28]. Multidisciplinary primary care teams and collaboration among different disciplines in multiple FFF-related activities are central to the FFF approach. Geriatric expertise is easily accessible by close involvement of elderly care physicians and geriatric nurses. Older persons’ (healthcare) needs are discussed in multidisciplinary consultation at least once a year. Individualized care plans include reported problems and (healthcare) needs, tailored (self-management) interventions, plans for multidisciplinary follow-up and evaluation. The care plan is discussed with the older person during a home visit by the practice nurse, homecare nurse or geriatric nurse. The care plan is then tailored to the person’s needs and wishes. Follow-up of older patients is arranged by a multidisciplinary team of (healthcare) professionals and an appointed case manager, who coordinates and evaluates the process, and provides support in goal setting and self-management. Older patients’ medication use is examined at least annually by GPs, elderly care physicians or pharmacists and discussed with the patients and their informal caregivers or relatives. Box 1 illustrates the application of the FFF approach. Further details on the components of the FFF approach have been published elsewhere [22, 24].

**Table 1:** Activities related to the FFF integrated primary care approach

FFF related activities	Explanation	Disciplines involved	Mean time per patient
Selection of patients	Selecting patients that are eligible for proactive frailty screening	GP or practice nurse	5 min
Proactive frailty screening	Home visit for administering the Tilburg Frailty Indicator (TFI) to assess frailty. Consultation with the patient and reporting needs and problems based on the SFSPC-model, i.e., model for reporting on Somatic, Functional, Social, Psychological, and Communicative indications	Practice nurse, geriatric nurse, or homecare nurse	90 min
Feedback information	Feedback information about the screening (e.g., TFI score) and problem analysis (SFSPC-model) to the GP and elderly care physician. First draft of individualized care plan for the patient	Practice nurse, geriatric nurse, or homecare nurse	100 min
Multidisciplinary consultation	Discussing the older patient in multidisciplinary consultation in the GP practice. Discussion of screening, problems listed according to SFSPC-model, possible (self-management) interventions, and involvement of (healthcare) professionals	*In general* GP Practice nurse Homecare nurse Elderly care physician Geriatric nurse *Frequently involved* Physiotherapist, occupational therapist and/or social worker	On average patients are discussed once or twice per year for 15 min
Individualized care plan	Definitive version of the individualized care plan is established, including (self-management) interventions discussed in multidisciplinary consultation	Practice nurse, geriatric nurse, or homecare nurse	10 min
Medication review	Older persons’ medication use is examined in a medication review	GP, pharmacist, or elderly care physician	10 min
Multidisciplinary follow-up	Individual follow-up of patients by a multidisciplinary team of (healthcare) professionals. A case manager is responsible for coordination and evaluation of the follow-up. An elderly care physician and geriatric nurse can provide geriatric expertise	Involvement of (healthcare) professionals based on the needs and wishes of the patient and can include, but are not limited to, practice nurses, physiotherapists, medical specialists, social workers, and so on	4 to 10 h

**Box 1 A case of a frail older person participating in the FFF approach**

Mr. Buys is 82 years old and has always lived in his parental home in the countryside near Roosendaal. His wife passed away 2 years ago and his two sons live and work in the capital city, Amsterdam. A diabetes check-up by his practice nurse raised alarm regarding Mr. Buys’ physical and social well-being. In response, the practice nurse screened Mr. Buys for frailty during a home visit; his TFI score was 8. In addition, Mr. Buys reported problems in the somatic, functional, and social domains. It became apparent that Mr. Buys misses having people around him and experiences problems in his daily life due to fatigue and difficulty in walking. He explained to the practice nurse that he lacks contact with his social network. After discussion with the GP and elderly care physician, a preliminary individualized care plan was established and Mr. Buys’ case was discussed in multidisciplinary consultation. Based on Mr. Buys’ reported problems and needs, a physiotherapist, geriatric nurse, and social worker were included in the multidisciplinary team, along with the practice nurse, GP, and elderly care physician. The geriatric nurse was appointed as Mr. Buys’ case manager (responsible for, e.g., discussing the (self-management) interventions that were proposed in the multidisciplinary consultation and adjusting the care plan to his wishes). The elderly care physician examined Mr. Buys’ medication use and arranged a home visit to evaluate his diabetes management. The physiotherapist visited Mr. Buys regularly to improve his physical functioning and to minimize fall risk through, e.g., walking and balance exercises. The case manager discussed several options to improve Mr. Buys’ social contact and independence. He decided to visit a day care center twice a week to be involved in meaningful activities and contact with older persons in his area of residence. The geriatric nurse contacted Mr. Buys (by home visit or telephone) and evaluated his follow-up regularly.

**Cost-utility and cost-effectiveness analyses**

The economic evaluation of the FFF approach consisted of a cost-utility analysis (CUA) and a cost-effectiveness analysis (CEA) from a healthcare perspective with a time horizon of 12 months. Costs and effects were measured at baseline (T0) and 12 months (T1). Trained interviewers administered questionnaires during in-home interviews to collect data regarding healthcare utilization and outcomes. The incremental cost-utility ratio (ICUR) and the incremental cost-effectiveness ratio (ICER) represent the difference in mean total costs adjusted for baseline costs between the intervention and control groups in the numerator and the difference in mean effectiveness adjusted for baseline effectiveness in the denominator.

**Outcomes and measures**

The primary outcome of the CUA was quality-adjusted life-years (QALYs). The validated EuroQol (EQ-5D-3L) was used as a preference-based health status measure to estimate utilities in the QALY measure [31, 32]. The descriptive system of the EQ-5D measures health-related quality of life in five health dimensions (mobility, self-care, usual activities, pain/discomfort, and anxiety/depression) on a three-point scale (1 = no problems, 2 = some or moderate problems, 3 = severe problems), resulting in 243 distinct health states [31, 32]. The EQ-5D health states were transformed into utility scores using the Dutch EQ-5D tariffs. Utilities based on the Dutch tariff range from − 0.33 to 1 (< 0 = health state considered worse than death, 0 = death or health state regarded to be equivalent to death, 1 = full health) [33]. For the CEA, the primary outcome was subjective well-being, measured with the validated short form of the Social Production Function Instrument for the Level of well-being (SPF-ILs) [34]. This 15-item instrument assesses whether five instrumental goals (comfort, stimulation, status, behavioral confirmation, and affection) are met in order to optimize universal goals of social and physical well-being [2, 34–36]. Mean SPF-ILs scores range from 1 to 4, with higher scores indicating greater subjective well-being [34]. EQ-5D and SPF-ILs outcomes were measured among frail older patients in the intervention and control groups at T0 and T1.

**Healthcare utilization and costs**

Total costs of intervention and control care at T0 and T1 were estimated as the summation of resources used multiplied by prices or valuations. Volumes of healthcare utilization were determined by the administration of resource use questionnaires during in-home interviews at T0 and T1. Frail older persons reported the types and frequencies (e.g., days of hospitalization or number of visits to the GP) of care they had received. To determine volumes of resource use, the following data were collected: numbers of GP consultations, out-of-hours GP consultations (i.e., at home or in the care clinic on evenings/nights/weekends), admissions to hospital/nursing home/home for the elderly, and visits to the physiotherapist/exercise therapist/psychologist/psychiatrist/social worker/medical specialist; and types of homecare service (i.e., household activities, personal care and nursing care at home), and elderly daycare or daycare treatment received. We also included costs related to the purchase of assistive aids (e.g., wheelchair) and in-home modifications as patient-related costs. Intervention costs included all costs related to the FFF activities, i.e., selection of patients, proactive screening for frailty, provision of feedback information, multidisciplinary consultation, individualized care plan development, medication review, and follow-up of frail older persons (see Table 1). We estimated the average amount of time spent on intervention related activities per patient by (a combination of) different healthcare professionals involved. Information for this estimation was collected by the FFF project leader and was based on registers of the contact persons for GP practices, minutes from multidisciplinary consultations, and observations made during frailty screening. The healthcare professionals involved in FFF-related activities differed among GP practices and frail older patients, due to the compositions of the practice teams, (healthcare) disciplines with services accessible in the region, and tailoring of the FFF approach to the wishes and needs of individual patients. FFF follow-up involved healthcare utilization (e.g., consultation with the practice nurse or social worker). To avoid duplicate inclusion of costs, we included such service use in the healthcare costs, and not in the intervention costs. Only consultations with healthcare professionals that were not registered on the resource use questionnaires were included in the intervention costs. Study-related activities and costs were excluded. We did not consider costs related to the training of involved healthcare professionals.

Healthcare resource volumes were valued using the Dutch manual for costing in healthcare [37]. Volumes of resource use were multiplied by standardized cost prices per unit of resource use (in euros) to estimate costs. Prices were inflated to 2015 (reference year) using the general consumer price index of 0.6% (Statistics Netherlands). When standardized costs per unit of resource use were unavailable, we estimated costs using true economic costs in the year 2015. To estimate costs related to out-of-hour GP consultations, we used the true economic weighted mean costs for this service in western North Brabant Province provided by the Dutch Healthcare Authority. Average expenditures based on Internet sources and expenditures obtained in previous research using the same resource use questionnaire were used to value purchased assistive aids and in-home modifications [38]. Annual depreciation costs were calculated according to the annuity method [37]. Intervention costs were based on the average amount of time invested per FFF element and hourly wages of the professionals involved, with proportional time investment applied when more than one professional was involved.

**Missing data**

Results are presented with and without imputation of missing values. Missing values were imputed according to the type of parameter (cost volume, utility, QALYs, SPF-ILs score), time point (T0, T1) and, for T1, reason for drop-out (see Fig. 1). Missing cost volumes at T0 were imputed with the mean cost volume of the specific service for the intervention group or control group at T0. The imputation of QALYs at T0 depended on the number of missing EQ-5D domains. When only one EQ-5D domain score was missing, the EQ-5D utility score was imputed using the median utility score of other persons in the same (intervention or control) group who had the same scores on the non-missing EQ-5D domains. When no participant in the same group had the same scores or more than one domain was missing, the missing utility score was replaced with the median utility score for the respective group. The mean SPF-ILs score was calculated when at least 10 of the 15 items were reported. Missing values at T0 were replaced with the mean SPF-ILs score in the respective group at T0. Missing costs, utilities, QALYs and SPF-ILs scores for participants in the intervention and control groups at T1 were imputed the same way as at T0. Missing costs and effects on T1 questionnaires of older persons that were lost to follow-up in the intervention group and control group between T0 and T1 (*n* = 50 and *n* = 56 respectively; see Fig. 1) were imputed as follows. Based on registrations of case managers and GPs, we estimated the number of months that a person lived at home, lived in a nursing/elderly home, and the number of months lost due to mortality. For each older person that was lost to follow-up, missing costs at T1 were imputed with the sum of (1) the number of months the person lived at home multiplied by the mean monthly healthcare costs (excluding the costs of nursing/elderly home admission) in the respective group at T1, and when applicable, (2) the number of months the person lived in a nursing/elderly home multiplied by monthly costs of nursing/elderly home admission, and (3) costs were set at zero from the month a participant died during the follow-up period. For persons for whom nothing further was known (*n *= 11), we used the mean healthcare costs in the respective group at T1. Missing QALYs at T1 were replaced with the sum of (1) the number of months a person lived at home multiplied by the median utility score at T1, and (2) the number of months a person lived in a nursing/elderly home multiplied by the utility score of 0.5 [39], and (3) a utility score of 0 was assigned from the month a person died. For persons for whom nothing further was known (*n *= 11), we used the QALYs in the respective group at T1. Finally, missing SPF-ILs values were imputed with the mean SPF-ILs group score at T1 due to the lack of SPF-ILs norm values. Additional file 1: Table S1 outlines the number of participants with missing data.

**Statistical analyses**

We assessed differences in baseline characteristics between the intervention and control groups using independent samples *t*-tests (for continuous variables with approximately normal distributions) and Chi squared tests (for categorical variables). Unadjusted differences in mean SPF-ILs scores and QALYs between groups were assessed using independent samples *t*-tests. Unadjusted differences in mean SPF-ILs scores and QALYs over time within each group were assessed using paired sample *t*-tests. Furthermore, these univariate analyses were complemented with multilevel analyses (linear mixed-effects models) to investigate effectiveness of the FFF approach. Multilevel models are considered appropriate for investigating relationships in data sets with continuous dependent variables and a clustered structure of the data (persons within GP practices) [40]. A random intercept was used on the level of the individual GP practices. Outcome estimates in the multilevel analyses were adjusted for baseline values of the respective outcome variable, background variables (i.e., age, sex, marital status, educational level, frailty score and multimorbidity) and control/intervention group. We performed the multilevel models (with QALYs and well-being as outcome estimates) using data with and without imputation of missing values.

Volumes of healthcare utilization were presented as means (and corresponding standard deviations; SDs) per service use category. Differences in costs between groups were tested using Mann–Whitney *U*-tests (skewed data) and independent samples *t*-tests (for mean values). Differences in costs over time within each group were assessed using related-samples Wilcoxon signed rank tests and paired sample *t*-tests.

Furthermore, propensity score matching was used to deal with potential different distributions of covariates between the intervention and control groups at baseline [41]. According to Indurkhya, Mitra, and Schrag [41], the propensity score is considered the probability that a person is assigned to the intervention group conditional on the person’s covariate information. For each individual person, the propensity to be part of the intervention group was estimated using a binary logistic regression model predicting assignment to the intervention group from baseline covariates. The covariates in the first logistic regression model (Model 1) were age, sex, marital status, educational level, frailty score, and multimorbidity. Next to these covariates, we also included baseline SPF-ILs, QALYs and costs in the second logistic regression model (Model 2). We then compared observed outcomes between intervention and control groups conditional on the propensity matched scores [42].

We performed nonparametric bootstrapping (percentile method) to generate 1500 samples from the original sample of 232 matched pairs. Predicted ICERs and ICURs were depicted on cost-effectiveness planes to show uncertainty therein. A statistical significance level of 5% (two-sided) was used in the analyses. All statistical analyses were performed with IBM SPSS Statistics version 24.

## Results

Table 2 shows the background characteristics of the study population at baseline. In total, 72.4% of participants were female, 41.8% had a low educational level, and 94.4% were considered to be frail according to the TFI (mean TFI score, 7.38) in both groups. At baseline, compared with participants in the control group, older persons in the intervention group were significantly less often single (*p *< 0.05). No significant difference in mean age or the proportion of older persons with multimorbidity was observed between the groups.

**Table 2:** Background characteristics of older persons in the two study groups at baseline

	Care as usual (*n* = 232)	FFF approach (*n* = 232)
Characteristics		
Age	82.41 (5.16)	82.45 (5.44)
Sex (female)	168 (72.4%)	168 (72.4%)
Marital status (single)	160 (69.0%)	134 (57.8%)*
Education (low)	97 (41.8%)	97 (41.8%)
Frailty score (TFI)	7.38 (2.39)	7.38 (2.40)
Frail (TFI score ≥ 5)	219 (94.4%)	219 (94.4%)
Multimorbidity (≥ 2 conditions)	208 (89.7%)	215 (92.6%)

Values are presented as mean (SD) or number (%)

TFI: Tilburg Frailty Indicator (range 0–15)

Independent samples *t*-tests or Chi squared tests

*p < 0.05 (two-tailed)

Table 3 shows the mean QALYs (with utilities based on the EQ-5D) and mean well-being scores (SPF-ILs) at T0 and T1 using the imputed dataset. Independent samples *t*-tests showed no statistically significant differences in QALYs between groups at T0 or T1 (univariate analysis). Paired sample *t*-tests showed a statistically significant improvement in QALYs over time in the control group (∆0.05; *p *< 0.05), but not in the intervention group (∆0.04; *p *= 0.07). Without imputation of missing values, the data also showed a significant improvement in terms of QALYs in the intervention group over time (paired sample *t*-test, ∆0.05; *p *< 0.05). Well-being did not differ significantly at T0 or T1 between the control and intervention groups, or over time in either group. Additional file 2: Table S2 displays the mean QALYs and SPF-ILs results of the univariate analyses based on data without imputation of missing values. Analyses based on data of matched participants, i.e., pairs with complete data, yielded comparable findings; independent samples *t*-tests showed no significant differences in mean QALYs and mean well-being scores between the groups at T0 and T1 (see Additional file 3: Tables S4–S7).

**Table 3:** Well-being and QALYs at baseline (T0) and 12 months (T1)

	Care as usual (*n* = 232)	FFF approach (*n* = 232)
Outcome measures		
Well-being (SPF-ILs)		
T0	2.62 (0.50)	2.63 (0.49)
T1	2.67 (0.49)	2.59 (0.46)
QALYs (utilities based on EQ-5D-3L)		
T0	0.66 (0.24)	0.63 (0.26)
T1	0.71 (0.20)*^,a^	0.67 (0.24)

Values are presented as mean (SD)

SPF-ILs: Social Production Function Instrument for the Level of well-being short (range 1–4); EQ-5D-3L: five-dimensional three-level EuroQol (range for utilities, − 0.33 to 1)

Data from univariate analyses after imputation of missing values

Paired sample *t*-tests or independent samples *t*-tests

*p < 0.05 (two-tailed)

^a^ Significant improvement in QALYs in the control group over time based on paired data

Multilevel analyses of SPF-ILs scores adjusted for background variables and baseline values showed a small but significant difference between the intervention group and control group for well-being at follow-up, in favor of the control group (− 0.09 (with imputation) and − 0.10 (without imputation)). No significant differences between the groups in terms of QALYs were observed (− 0.03 (with imputation) and − 0.02 (without imputation)) (Additional file 4: Tables S8–S11). Regression analyses to investigate multivariable relationships among the variables yielded comparable results as the multilevel analyses (details not shown). The multilevel analyses were redone for the propensity score matched group which showed a significant difference between the groups for well-being in favor of the control group [(− 0.09 (with imputation) and − 0.10 (without imputation)]. We found no significant differences in QALYs between the intervention group and control group [− 0.03 (with imputation) and − 0.02 (without imputation)]. For details see Additional file 5: Tables S12–S19.

For the imputed dataset, mean total costs were 7717 euros (SD, 9824 euros) in the control group and 9182 euros (SD, 11,754 euros) in the intervention group at baseline (independent samples *t*-test, *p *= 0.15; Mann–Whitney *U*-test, *U* = 28,618.50, t = 1.18, *p *= 0.24; Table 4). At 12 months, mean total costs were significantly higher in the intervention group (11,659 [SD, 14,600] euros; including intervention costs) than in the control group (8902 [SD, 11,227] euros) (independent samples *t*-test, *p *< 0.05; Table 5). In addition, differences in the median total costs at follow-up were statistically significant (Mann–Whitney *U*-test, *U* = 29,952.00, t = 2.11, *p *< 0.05). The mean total costs increased significantly over time in the intervention group (paired sample *t*-test, *p *< 0.05), but not in the control group (paired sample *t*-test, *p *= 0.14). The difference in median costs between T0 and T1 was significant in the intervention group (related-samples Wilcoxon signed rank test, t = 3.18, *p *< 0.05) and in the control group (related-samples Wilcoxon signed rank test, t = 2.34, *p *< 0.05). Based on the data without imputation of missing values, no statistically significant differences in total costs between the control and intervention groups were found at baseline (independent samples *t*-test, *p* = 0.15; Mann–Whitney *U*-test, *U* = 17,165.50, t = 0.60, *p* = 0.55) and 12 months (independent samples *t*-test, *p* = 0.09; Mann–Whitney *U*-test, *U* = 14,375.50, t = 1.09, *p* = 0.28). For details see Additional file 2: Table S3. In addition, univariate analyses for the propensity score matched group yielded comparable results; for the imputed dataset mean total costs were significantly higher in the intervention group compared with the control group at 12 months. Based on data without imputation of missing values, no significant differences in mean total costs between groups were observed at both time points. Univariate analyses based on data of matched participants, i.e., pairs with complete data, showed no significant difference in mean total costs between the groups at baseline and follow-up. See Additional file 3: Tables S4–S7.

**Table 4:** Healthcare use and costs (in euros) per patient per year in the intervention and control groups at baseline

	Care as usual (*n* = 232)		FFF approach (*n* = 232)	
	Mean use^a^ (SD)	Total costs (SD) in €	Mean use^a^ (SD)	Total costs (SD) in €
Healthcare				
Hospital (days)	1.87 (4.90)	893.66 (2327.38)	1.76 (5.53)	845.29 (2634.78)
Consultations with the GP	3.51 (2.93)	116.43 (94.87)	3.80 (3.90)	126.25 (126.85)
Consultations out-of-hours GP	0.23 (0.67)	22.12 (63.01)	0.28 (0.70)	26.74 (65.77)
Professional homecare (hours per week)				
Household activities (homecare or personal budget)	1.58 (1.77)	1780.95 (1967.73)	1.67 (1.76)	1887.95 (1955.74)
Household activities (private)	0.52 (1.20)	582.04 (1320.02)	0.59 (1.80)	664.86 (1988.32)
Personal care	0.88 (1.89)	2315.93 (4766.32)	1.04 (2.22)	2731.71 (5583.19)
Nursing care	0.28 (1.48)	1085.97 (5512.92)	0.29 (1.57)	1108.73 (5757.76)
Care home (weeks)	0.11 (0.96)	129.16 (1128.74)	0.13 (1.37)	151.08 (1613.16)
Nursing home (weeks)	0.07 (0.71)	77.49 (838.43)	0.14 (1.09)	162.03 (1285.98)
Elderly day care (days per week)	0.03 (0.27)	213.34 (1889.62)	0.09 (0.49)	606.89 (3397.51)
Day care treatment (days per week)	0.0 (0.0)	0.0 (0.0)	0.03 (0.23)	375.02 (3269.36)
Physiotherapist (consultations)	6.77 (15.34)	224.68 (506.64)	6.75 (16.22)	224.06 (537.29)
Exercise therapist (consultations)	0.28 (1.94)	9.41 (65.98)	0.72 (5.77)	24.75 (195.62)
Medical specialist (consultations)	2.71 (4.58)	248.02 (408.44)	2.33 (3.36)	213.07 (301.12)
Social worker (sessions)	0.09 (1.03)	6.05 (66.81)	0.02 (0.21)	1.14 (13.56)
Psychologist or psychiatrist (sessions)	0.06 (0.47)	3.99 (29.69)	0.25 (1.30)	15.96 (83.10)
Assistive aids and in-home modifications				
Wheelchair	0.03 (0.18)	1.63 (8.65)	0.04 (0.20)	2.04 (9.63)
Alarm system	0.06 (0.25)	1.61 (6.14)	0.06 (0.23)	1.40 (5.74)
Wheeled walker	0.09 (0.29)	1.86 (5.91)	0.13 (0.34)	2.66 (6.91)
Stairlift	0.0 (0.0)	0.0 (0.0)	0.02 (0.15)	6.71 (45.30)
Adjusted doorsteps	0.004 (0.07)	0.08 (1.23)	0.02 (0.13)	0.32 (2.44)
Adjusted bathroom	0.09 (0.29)	3.31 (10.24)	0.11 (0.31)	3.76 (10.84)
Mean total costs^b^		7717.72 (9824.92)		9182.42 (11,754.75)

^a^Means (SDs) were calculated including persons without healthcare utilization

^b^Mean total costs calculated after imputation of missing healthcare costs

**Table 5:** Healthcare use and costs (in euros) per patient per year in the intervention and control groups at 12 months

	Care as usual (*n* = 176)		FFF approach (*n* = 182)	
	Mean use^a^ (SD)	Total costs (SD) in €	Mean use^a^ (SD)	Total costs (SD) in €
Healthcare				
Hospital (days)	2.66 (6.92)	1275.14 (3305.59)	2.14 (9.55)	1026.89 (4546.80)
Consultations with the GP	3.57 (3.41)	118.61 (111.78)	3.83 (3.44)	127.14 (113.30)
Consultations out-of-hours GP	0.25 (0.88)	24.23 (82.89)	0.22 (0.60)	21.05 (56.49)
Professional homecare (hours per week)				
Household activities (homecare or personal budget)	1.16 (1.50)	1302.25 (1671.23)	1.58 (1.63)	1772.57 (1816.37)
Household activities (private)	0.76 (1.37)	850.15 (1520.05)	0.46 (1.21)	520.28 (1344.72)
Personal care	0.92 (1.91)	2393.81 (4928.75)	1.37 (2.83)	3574.16 (7270.45)
Nursing care	0.11 (0.50)	432.95 (1871.74)	0.31 (1.33)	1182.55 (4976.57)
Care home (weeks)	0.20 (1.27)	236.61 (1496.48)	0.17 (1.18)	197.18 (1387.62)
Nursing home (weeks)	0.09 (0.81)	102.85 (962.21)	0.28 (2.15)	330.47 (2524.32)
Elderly day care (days per week)	0.05 (0.37)	358.45 (2624.88)	0.05 (0.33)	350.48 (2361.89)
Day care treatment (days per week)	0.02 (0.23)	246.11 (3264.99)	0.04 (0.30)	641.70 (4244.80)
Physiotherapist (consultations)	6.01 (10.89)	199.39 (361.68)	8.64 (18.07)	286.74 (594.85)
Exercise therapist (consultations)	0.31 (3.48)	10.75 (118.69)	1.36 (9.63)	46.36 (327.42)
Medical specialist (consultations)	2.65 (4.07)	242.74 (371.38)	2.25 (3.17)	227.60 (287.96)
Social worker (sessions)	0.06 (0.45)	3.72 (29.43)	0.45 (4.06)	29.59 (263.51)
Psychologist or psychiatrist (sessions)	0.04 (0.38)	2.58 (24.20)	0.22 (1.20)	13.95 (76.65)
Assistive aids and in-home modifications				
Wheelchair	0.01 (0.11)	0.54 (5.03)	0.03 (0.18)	1.57 (8.47)
Alarm system	0.06 (0.23)	1.41 (5.78)	0.08 (0.28)	2.06 (6.87)
Wheeled walker	0.07 (0.25)	1.40 (5.20)	0.09 (0.29)	1.93 (6.00)
Stairlift	0.01 (0.11)	3.54 (33.09)	0.02 (0.13)	5.16 (39.74)
Adjusted doorsteps	0.02 (0.15)	0.42 (2.79)	0.0 (0.0)	0.0 (0.0)
Adjusted bathroom	0.09 (0.28)	2.97 (9.76)	0.03 (0.16)	0.96 (5.72)
Mean total costs^b^		8902.06 (11,227.42)		11,426.21 (14,600.79)
Mean total intervention costs		n/a		233

^a^Means (SDs) were calculated including persons without healthcare utilization

^b^Mean total costs calculated after imputation of missing healthcare costs including persons lost to follow-up between T0 and T1 (*n* = 50 in the intervention group and *n* = 56 in the control group)

**Fig. 2:**
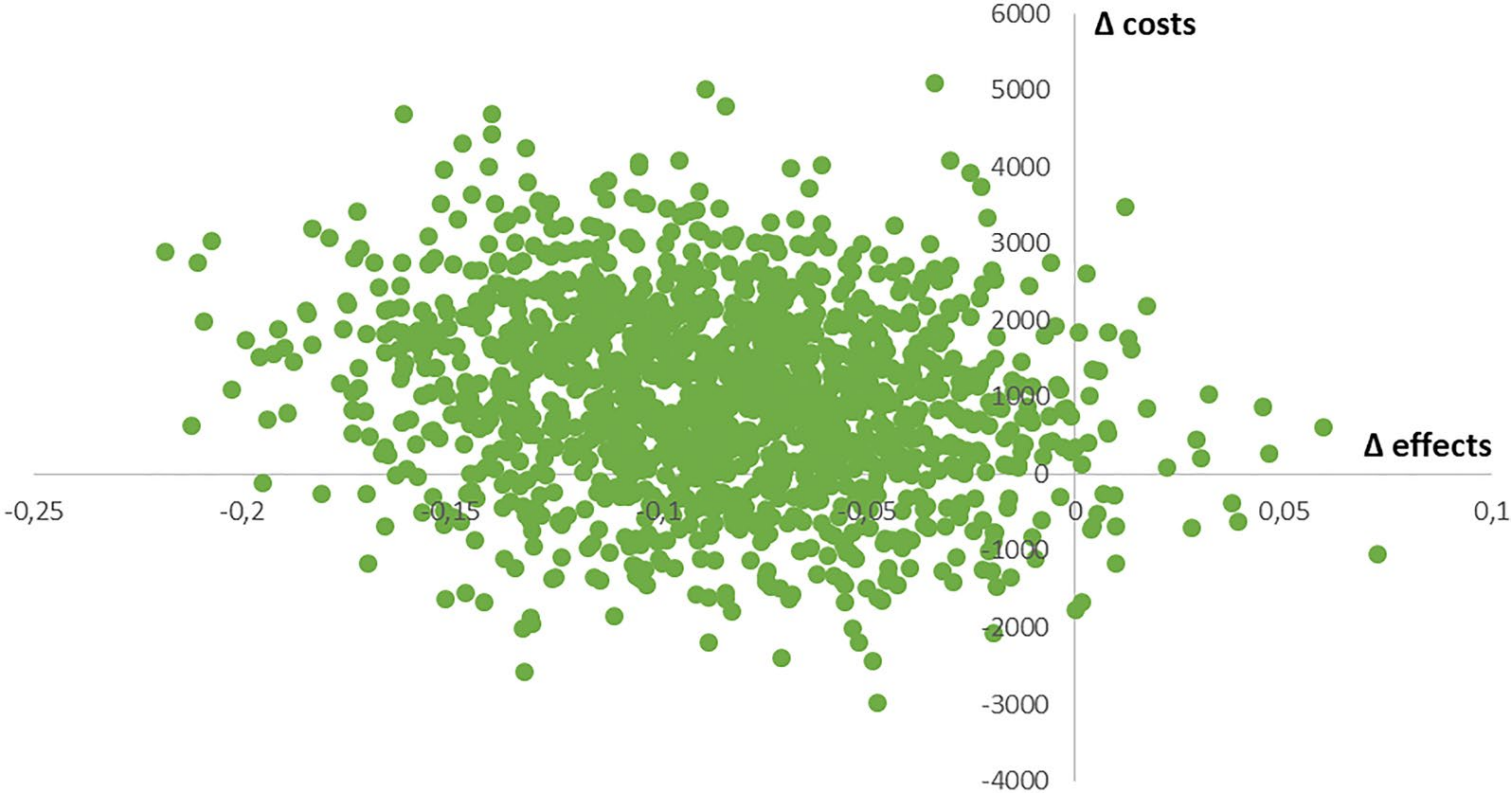
Cost-effectiveness plane for costs (in euros) versus effects (SPF-ILs; range 1–4) adjusted for baseline differences; data after imputation of missing values

**Fig. 3:**
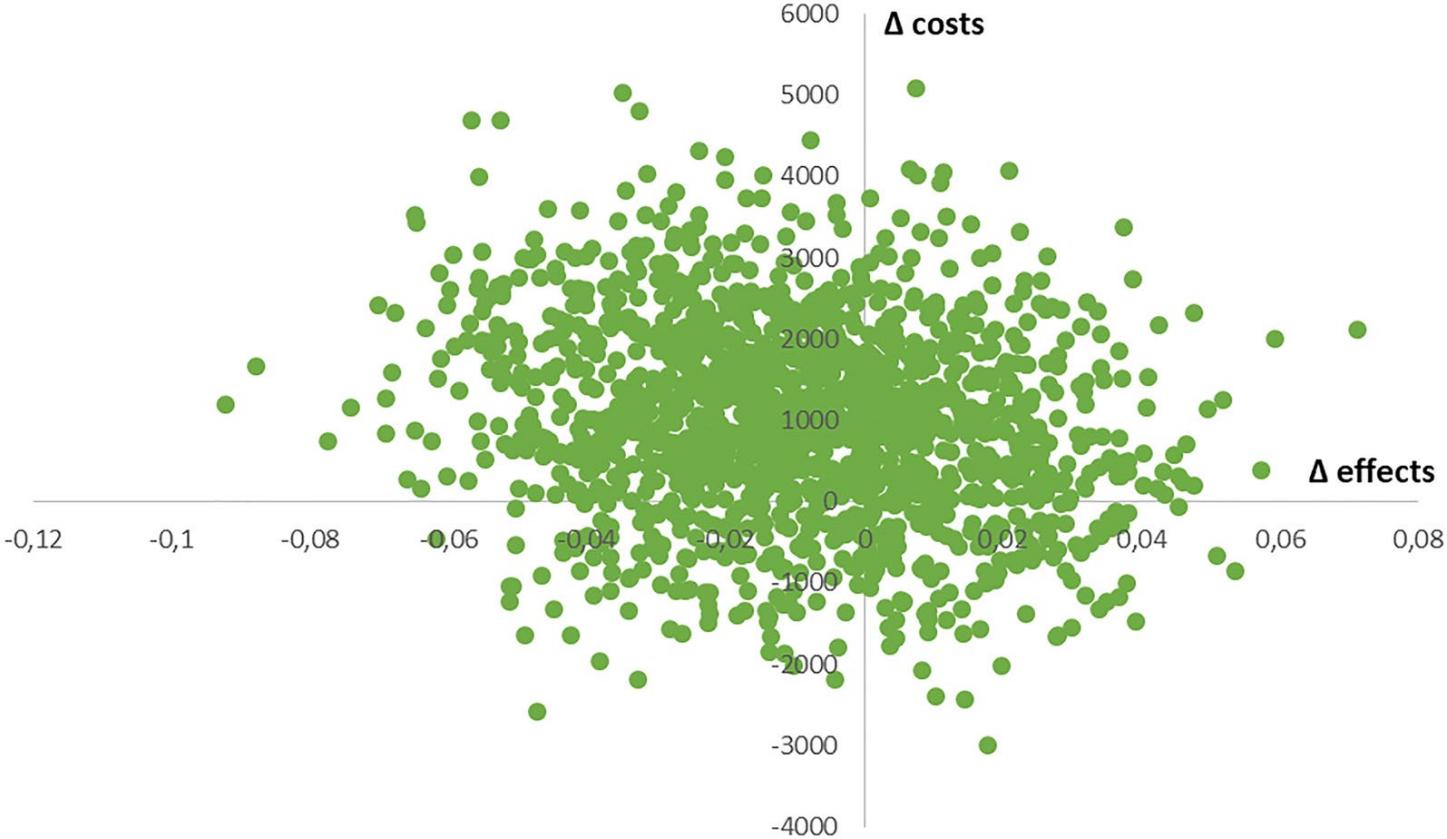
Cost-effectiveness plane for costs (in euros) versus effects (QALYs; range − 0.33 to 1) adjusted for baseline differences; data after imputation of missing values

Using the imputed dataset, estimated differences in effectiveness and costs were both in favor of usual care, producing an ICER of − 14,788 euros per SPF-ILs point and an ICUR of − 126,711 euros per QALY, indicating the FFF approach is inferior in both approaches. In Fig. 2 (cost-effectiveness plane for costs versus effects in terms of well-being; SPF-ILs), 0.9% of all bootstrapped ICERs appear in the southeast quadrant (dominance; FFF approach is more effective and less costly), 78.9% appear in the northwest quadrant (inferiority; FFF intervention is more expensive and less effective), 1.5% appear in the northeast quadrant (FFF intervention is more effective, but also more expensive) and 18.7% appear in the southwest quadrant (FFF intervention is less costly, but also less effective). The probability that the FFF approach is cost-effective ranges between 0.9% and 21.1%, depending on the cost-effectiveness ratio a decision maker could apply for policy decisions. In Fig. 3 (cost-effectiveness plane for costs versus effects in terms of QALYs), 9.0% of bootstrapped ICURs are located in the southeast quadrant, 54.4% appear in the northwest quadrant, 26.1% are located in the northeast quadrant, and 10.5% are located in the southeast quadrant. The probability that the FFF approach is cost-effective ranges between 9.0% and 45.6%, depending on the cost-effectiveness ratio applied.

Although different analyses (e.g., univariate, multilevel, and propensity score matched analyses, with and without imputation) showed slightly different results with respect to estimated costs and effects, the data suggest that the FFF approach is most likely not cost-effective compared with usual primary care in the Netherlands in terms of well-being and QALYs over a 12 month-period, irrespective of analytical approach and method of handling missing values.

## Discussion

The results of our economic evaluation indicate that proactive, integrated care for community-dwelling frail older persons as provided in the FFF program is most likely not a cost-effective initiative compared with usual primary care in the Netherlands, in terms of well-being and QALYs over a 12-month period. Our results are in line with outcomes of other studies investigating the cost-effectiveness of integrated care for frail older persons in the primary care setting in the Netherlands [43]. The comparability of integrated care programs and evaluation studies is limited due to differences in study populations, interventions and outcomes [16].

One explanation for the lack of effect may be the conceivably small difference between the FFF approach and usual primary care services in the Netherlands. There are indications that reforms in the primary care system in the Netherlands resulted in developments in the control GP practices to improve their care delivery. Although these practices did not provide care and support according to the FFF approach, several control GP practices implemented interventions, such as systematic follow-up of older adults and multidisciplinary consultation, during the study period [22]. In addition, the lack of effectiveness of complex interventions may be partly due to failure to (fully) implement the programs as intended [44]. Indeed, we have found suboptimal implementation of intervention components in GP practices organizing care according to the FFF approach [22]. Most interventions, and especially complex care programs like the FFF approach, require extensive time and effort to achieve full implementation [45, 46]. We noted differences among intervention GP practices with respect to the implementation and execution of the FFF program, including differences in the selection of older persons for proactive screening, the (number of) professionals involved in screening procedures, the organization of multidisciplinary consultations (e.g., frequency, number of patients discussed, (type of) professionals involved), and the organization of long-term follow-up of frail older persons [22]. These differences may obscure the added value of the FFF approach in terms of QALYs and well-being. Analyses based on matched participants of intervention GP practices with a high degree of implementation of (FFF-related) interventions (i.e., practices that implemented more interventions than average) [22], showed that the mean SPF-ILs score was higher, indicating greater subjective well-being, compared with participants in other intervention GP practices (Additional file 6: Tables S20–S23). Therefore, the degree of implementation may have an effect on effectiveness of complex interventions like the FFF approach. For a detailed description of implemented (FFF-related) interventions in the GP practices see Vestjens, Cramm and Nieboer [22]. Even with optimal implementation of such interventions, clinically meaningful improvement in outcomes is not guaranteed in the short term [45]. The length of the study period, being 12 months, may have been too short to detect improvements in older persons’ outcomes [20]. Especially in the short term, variations in costs and effects can be expected [47]. Patterns of healthcare utilization show, for example, a substantial increase in primary and hospital care utilization in frail older persons [9]. Consequently, the identification of frailty and introduction of interventions to postpone or prevent a decline into worse health states [48] may result in higher healthcare costs in the short term, but might reduce use of more expensive healthcare services and adverse outcomes in the long term [9]. Another explanation might be related to the heterogeneity of the population of older persons considered to be frail. No consensus has been reached about the conceptualization and measurement of frailty in older persons. Major approaches include the frailty phenotype, which focuses on physical aspects of frailty [49, 50], and a multidimensional approach to frailty including, for example, physical, social, and psychological factors [51]. Although we used a multidimensional approach to assess frailty in this study, Looman and colleagues [52] showed that distinction among domains of frailty does not fully capture its complexity. The TFI [30], which we used to measure (the degree of) frailty in older persons, does not discern among types of underlying problems in these domains or weigh different domains [52]. Researchers have suggested that the heterogeneity of frailty should be taken into account in the evaluation of integrated care programs [52], especially to better understand how interventions can be optimally aligned with different well-being needs of frail older persons [2].

### Strengths and limitations

One strength of this study is that we measured the subjective well-being of community-dwelling frail older persons along with health-related quality of life. QALY measures in economic evaluations are based predominantly on aspects of health-related quality of life alone. Care programs for older persons may also aim to improve non-health related domains of quality of life. Thus, the sole use of health-related quality of life measures in economic evaluations may not be appropriate, as it may not capture broader benefits of such interventions beyond health [53]. Consequently, Makai and colleagues [53] recommended the inclusion of well-being measures with health measures like the EQ-5D in economic evaluations of care programs for older persons. We did so, although the different perspectives did not lead to different recommendations regarding the preference of the FFF intervention. Another strength of our study is the quality of the data gathered. We used dedicated, trained interviewers who collected the data in face-to-face interviews during home visits. All interviewers lived in the western North Brabant Province, assuring a cultural fit, and had backgrounds in healthcare. Moreover, we used a detailed resource use questionnaire covering a wide range of healthcare categories to assess healthcare utilization at the individual level. We included care disciplines that are frequently not included in studies, such as paramedical (e.g., physiotherapy) and psychological care, which may have increased content validity. Our study also has several potential limitations. First, we used a quasi-experimental design, which is more susceptible to bias due to the absence of randomization [54]. To increase comparability of the intervention and control groups, we used one-to-one matching based on key covariables. Despite this effort, the control group contained significantly more single persons than did the intervention group. Moreover, we noted indications (based on interviews with healthcare professionals and (project) managers) of a strong motivation to organize care and support for the elderly population in some control GP practices. Professionals in these practices may have perceived that the FFF program would not add value to their usual care practices and were therefore perhaps especially eager to participate in the control group. Second, recall bias might have occurred due to the retrospective assessment of service use in the preceding 12 months. Under-reporting and over-reporting of effects have been found in previous research in which health service utilization was assessed retrospectively [55]. Unfortunately, we were not able to include administrative or registry data to complement the reported healthcare service use. Nonetheless, given the same data collection procedure in both groups, we have no indication that recall bias varied significantly between the intervention and control groups. Third, mean standard costs of the FFF program were estimated, instead of assessing intervention costs for individual participants. We attempted to avoid duplicate inclusion of costs by including service use related to the follow-up of older patients in the FFF context only in healthcare costs, and not in intervention costs. The implementation and execution of (elements of) the FFF approach differed among intervention GP practices [22]. However, results of sensitivity analyses in which intervention costs were varied to test the robustness of the estimated ICER and ICUR did not affect the overall recommendation regarding the preference of the FFF program. Fourth, despite recommendations [37], we were unable to collect data on informal care due to practical considerations. The impact of informal care costs on the mean total costs in the intervention and control groups remains unknown, although we found no indication (based on interviews with healthcare professionals, (project) managers, and frail older persons) of unequal distribution of informal care costs between groups. In addition, we did not account for medication costs in either group or intervention training and implementation costs in the FFF group. We have noted no indication that medication use differed between groups.

## Conclusions

Our study findings add to the current unconvincing body of evidence with respect to the cost-effectiveness of integrated primary care aimed at community-dwelling frail older persons. Future economic evaluations should use sufficiently long follow-up periods to assess durable costs and effects, adopt a societal perspective, and take into account the degree of implementation and the target population. Continued effort is required to unravel the black box of integrated care and find (cost-)effective (components of) programs for community-dwelling frail older persons.

## Additional files

**Additional file 1: Table S1.** Number of participants with missing data on the EQ-5D-3L, SPF-ILs and resource use questionnaire at T0 and T1 (total *n* = 464).

**Additional file 2: Table S2.** Well-being and QALYs at baseline (T0) and 12 months (T1) ***without*** data imputation. **Table S3.** Healthcare costs (in euros) at baseline (T0) and 12 months (T1) ***without*** data imputation.

**Additional file 3: Table S4.** Analyses based on matched participants, i.e., pairs with complete data on EQ-5D-3L and resource use, at T0 (*n* = 146 pairs) and at T1 (*n* = 111 pairs). **Table S5.** Analyses based on matched participants, i.e., pairs with complete data on SPF-ILs and resource use, at T0 (*n* = 145 pairs) and T1 (*n* = 111 pairs). **Table S6.** Analyses based on matched participants, i.e., pairs with complete data on EQ-5D-3L and resource use on ***both*** T0 and T1 (*n* = 71 pairs). **Table S7.** Analyses based on matched participants, i.e., pairs with complete data on SPF-ILs and resource use on ***both*** T0 and T1 (*n* = 70 pairs).

**Additional file 4: Table S8.** Multilevel analyses of well-being (SPF-ILs) at T1, after data imputation (*n* = 464). **Table S9.** Multilevel analyses of well-being (SPF-ILs) at T1, ***without*** data imputation (*n* = 354). **Table S10.** Multilevel analyses of QALYs at T1, after data imputation (*n* = 464). **Table S11.** Multilevel analyses of QALYs at T1, ***without*** data imputation (*n* = 355).

**Additional file 5: Table S12.** Multilevel analyses well-being, using propensity score matching (Model 1)^a^ and data imputation (*n* = 463). **Table S13.** Multilevel analyses well-being, using propensity score matching (Model 2)^a^ and data imputation (*n* = 459). **Table S14.** Multilevel analyses well-being, using propensity score matching (Model 1)^a^ and ***without*** data imputation (*n* = 353). **Table S15.** Multilevel analyses well-being, using propensity score matching (Model 2)^a^ and ***without*** data imputation (*n* = 349). **Table S16.** Multilevel analyses QALYs, using propensity score matching (Model 1)^a^ and data imputation (*n* = 463). **Table S17.** Multilevel analyses QALYs, using propensity score matching (Model 2)^a^ and data imputation (*n* = 459). **Table S18.** Multilevel analyses QALYs, using propensity score matching (Model 1)^a^ and ***without*** data imputation (*n* = 354). **Table S19.** Multilevel analyses QALYs, using propensity score matching (Model 2)^a^ and ***without*** data imputation (*n* = 351).

**Additional file 6: Table S20.** Analyses of participants of intervention GP practices with high degree of implementation and matched controls ***without*** data imputation. **Table S21.** Healthcare costs (in euros) of participants of intervention GP practices with high degree of implementation and matched controls ***without*** data imputation. **Table S22.** Analyses of participants of intervention GP practices with high degree of implementation and matched controls, after data imputation. **Table S23.** Healthcare costs (in euros) of participants of intervention GP practices with high degree of implementation and matched controls, after data imputation.

## Data Availability

The datasets generated and analyzed during the current study are not publicly available, but are available from the corresponding author on reasonable request.

## Abbreviations

CEA: cost-effectiveness analysis
CUA: cost-utility analysis
EQ-5D-3L: EuroQol (5 dimensions and 3 levels)
FFF: Finding and Follow-up of Frail older persons
GP(s): general practitioner(s)
ICER(s): incremental cost-effectiveness ratio(s)
ICUR(s): incremental cost-utility ratio(s)
QALY(s): quality-adjusted life-year(s)
SD: standard deviation
SFSPC-model: model for reporting on Somatic, Functional, Social, Psychological, and Communicative indications
SPF-ILs: Social Production Function Instrument for the Level of well-being short
TFI: Tilburg Frailty Indicator
