# Supplementary material for: Cost-effectiveness of a proactive, integrated primary care approach for community-dwelling frail older persons
Source: Cost Eff Resour Alloc. 2019 Jul 9;17:14. doi: 10.1186/s12962-019-0181-8 (PMC6617694; doi:10.1186/s12962-019-0181-8)
Supplement: Supplementary file 1 — Additional file 1: Table S1. Number of participants with missing data on the EQ-5D-3L, SPF-ILs and resource use questionnaire at T0 and T1 (total n = 464). [file 12962_2019_181_MOESM1_ESM.docx]

**ADDITIONAL MATERIALS**

**Additional file 1. Overview of missing data**

**Table S1** Number of participants with missing data on the EQ-5D-3L, SPF-ILs and resource use questionnaire at T0 and T1 (total *n* = 464)

|  | T0 |  |  | T1 |  |  |
| --- | --- | --- | --- | --- | --- | --- |
|  | EQ-5D-3L | SPF-ILs | Resource use | EQ-5D-3L | SPF-ILs | Resource use |
| Data completely missing^a^ | 3 | 4 | 0 | 108^c^ | 108^c^ | 106^c^ |
| Data partially missing^b^ | 1 | 4 | 100 | 0 | 1 | 30 |

SPF-ILs: Social Production Function Instrument for the Level of well-being short; EQ-5D-3L: five-dimensional three-level EuroQol. ^a^Data completely missing = none of the items in the questionnaires are answered. ^b^Data partially missing EQ-5D-3L = 1-4 missings of the 5 questions on the EQ-5D-3L; Data partially missing SPF-ILs = 6-14 missings of the 15 questions on the SPF-ILs; Data partially missing resource use = 1-21 missings of the 22 questions on the resource use instrument. ^c^Including persons lost to follow-up between T0 and T1 (total *n* = 106, see Fig. 1)
