# Supplementary material for: Cost-effectiveness of a proactive, integrated primary care approach for community-dwelling frail older persons
Source: Cost Eff Resour Alloc. 2019 Jul 9;17:14. doi: 10.1186/s12962-019-0181-8 (PMC6617694; doi:10.1186/s12962-019-0181-8)
Supplement: Supplementary file 2 — Additional file 2: Table S2. Well-being and QALYs at baseline (T0) and 12 months (T1) without data imputation. Table S3. Healthcare costs (in euros) at baseline (T0) and 12 months (T1) without data imputation. [file 12962_2019_181_MOESM2_ESM.docx]

**ADDITIONAL MATERIALS**

**Additional file 2. Outcomes and cost estimates without imputation of missing values**

**Table S2** Well-being and QALYs at baseline (T0) and 12 months (T1) ***without*** data imputation

|  |  | **Care as usual** | ***n*** | **FFF approach** | ***n*** |
| --- | --- | --- | --- | --- | --- |
| **Outcome measures** |  |  |  |  |  |
| Well-being (SPF-ILs) | T0 | 2.62 (0.50) | 230 | 2.63 (0.50) | 226 |
|  | T1 | 2.71 (0.53) | 176 | 2.60 (0.50) | 179 |
| QALYs  (utilities based on EQ-5D-3L) | T0 | 0.66 (0.25) | 230 | 0.63 (0.26) | 230 |
|  | T1 | 0.72 (0.21)*^a^ | 176 | 0.70 (0.25)*^a^ | 180 |

Values are presented as mean (SD)

SPF-ILs: Social Production Function Instrument for the Level of well-being short (range, 1-4); EQ-5D-3L: five-dimensional three-level EuroQol (range for utilities, -0.33-1)

Data from univariate analyses ***without*** imputation of missing values

**p* < 0.05 (two-tailed)

Paired sample *t*-tests or independent samples *t*-tests

^a^ Significant improvement in QALYs in the control group and intervention group over time based on paired data

**Table S3** Healthcare costs (in euros) at baseline (T0) and 12 months (T1) ***without*** data imputation

|  | **Care as usual** | ***n*** | **FFF approach** | ***n*** |
| --- | --- | --- | --- | --- |
| **Healthcare costs** |  |  |  |  |
| Mean total costs at T0^a^ | 7023.18 (9499.67) | 180 | 8635.90 (11,735.34) | 184 |
| Mean total costs at T1^a^ | 7798.13 (10,442.08) | 160 | 10,209.36 (14,598.36) | 168 |

^a^Means (SDs) were calculated including persons without healthcare utilization
