# Supplementary material for: Cost-effectiveness of a proactive, integrated primary care approach for community-dwelling frail older persons
Source: Cost Eff Resour Alloc. 2019 Jul 9;17:14. doi: 10.1186/s12962-019-0181-8 (PMC6617694; doi:10.1186/s12962-019-0181-8)
Supplement: Supplementary file 3 — Additional file 3: Table S4. Analyses based on matched participants, i.e., pairs with complete data on EQ-5D-3L and resource use, at T0 (n = 146 pairs) and at T1 (n = 111 pairs). Table S5. Analyses based on matched participants, i.e., pairs with complete data on SPF-ILs and resource use, at T0 (n = 145 pairs) and T1 (n = 111 pairs). Table S6. Analyses based on matched participants, i.e., pairs with complete data on EQ-5D-3L and resource use on both T0 and T1 (n = 71 pairs). Table S7. Analyses based on matched participants, i.e., pairs with complete data on SPF-ILs and resource use on both T0 and T1 (n = 70 pairs). [file 12962_2019_181_MOESM3_ESM.docx]

**ADDITIONAL MATERIALS**

**Additional file 3. Analyses based on matched participants**

**Table S4** Analyses based on matched participants, i.e., pairs with complete data on EQ-5D-3L and resource use, at T0 (*n* = 146 pairs) and at T1 (*n* = 111 pairs)

|  |  | **Care as usual** | **FFF approach** |
| --- | --- | --- | --- |
| QALYs  (utilities based on EQ-5D-3L) | T0 | 0.66 (0.24) | 0.63 (0.24) |
|  | T1 | 0.74 (0.20) | 0.71 (0.23) |
| Mean total costs^a^  (resource use questionnaire) | T0 | 6742.44 (9958.21) | 8829.61 (11,594.37) |
|  | T1 | 8330.41 (11,368.95) | 9029.57 (12,942.87) |

Values are presented as mean (SD); ^a^Means (SDs) were calculated including persons without healthcare utilization

EQ-5D-3L: five-dimensional three-level EuroQol (range for utilities, -0.33-1)

**p* < 0.05 (two-tailed)

Independent samples *t*-tests or Mann-Whitney *U*-tests

**Table S5** Analyses based on matched participants, i.e., pairs with complete data on SPF-ILs and resource use, at T0 (*n* = 145 pairs) and T1 (*n* = 111 pairs)

|  |  | **Care as usual** | **FFF approach** |
| --- | --- | --- | --- |
| Well-being (SPF-ILs) | T0 | 2.64 (0.49) | 2.64 (0.47) |
|  | T1 | 2.75 (0.53) | 2.62 (0.53) |
| Mean total costs^a^  (resource use questionnaire) | T0 | 6878.86 (10,038.26) | 8651.38 (11,287.80) |
|  | T1 | 8330.41 (11,368.95) | 9029.57 (12,942.87) |

Values are presented as mean (SD); ^a^Means (SDs) were calculated including persons without healthcare utilization

SPF-ILs: Social Production Function Instrument for the Level of well-being short (range, 1-4)

**p* < 0.05 (two-tailed)

Independent samples *t*-tests or Mann-Whitney *U*-tests

**Table S6** Analyses based on matched participants, i.e., pairs with complete data on EQ-5D-3L and resource use on ***both*** T0 and T1 (*n* = 71 pairs)

|  |  | **Care as usual** | **FFF approach** |
| --- | --- | --- | --- |
| QALYs  (utilities based on EQ-5D-3L) | T0 | 0.68 (0.24) | 0.68 (0.22) |
|  | T1 | 0.74 (0.21) | 0.73 (0.21) |
| Mean total costs^a^  (resource use questionnaire) | T0 | 6071.65 (9243.57) | 7435.85 (9709.72) |
|  | T1 | 7848.30 (11,386.60) | 9038.16 (13,728.47) |

Values are presented as mean (SD); ^a^Means (SDs) were calculated including persons without healthcare utilization

EQ-5D-3L: five-dimensional three-level EuroQol (range for utilities, -0.33-1)

**p* < 0.05 (two-tailed)

Independent samples *t*-tests or Mann-Whitney *U*-tests

**Table S7** Analyses based on matched participants, i.e., pairs with complete data on SPF-ILs and resource use on ***both*** T0 and T1 (*n* = 70 pairs)

|  |  | **Care as usual** | **FFF approach** |
| --- | --- | --- | --- |
| Well-being (SPF-ILs) | T0 | 2.74 (0.49) | 2.68 (0.48) |
|  | T1 | 2.78 (0.52) | 2.65 (0.53) |
| Mean total costs^a^  (resource use questionnaire) | T0 | 6107.91 (9305.23) | 7508.36 (9761.75) |
|  | T1 | 7952.83 (11,434.45) | 9135.14 (13,803.08) |

Values are presented as mean (SD); ^a^Means (SDs) were calculated including persons without healthcare utilization

SPF-ILs: Social Production Function Instrument for the Level of well-being short (range, 1-4)

**p* < 0.05 (two-tailed)

Independent samples *t*-tests or Mann-Whitney *U*-tests
