# Supplementary material for: Cost-effectiveness of a proactive, integrated primary care approach for community-dwelling frail older persons
Source: Cost Eff Resour Alloc. 2019 Jul 9;17:14. doi: 10.1186/s12962-019-0181-8 (PMC6617694; doi:10.1186/s12962-019-0181-8)
Supplement: Supplementary file 4 — Additional file 4: Table S8. Multilevel analyses of well-being (SPF-ILs) at T1, after data imputation (n = 464). Table S9. Multilevel analyses of well-being (SPF-ILs) at T1, without data imputation (n = 354). Table S10. Multilevel analyses of QALYs at T1, after data imputation (n = 464). Table S11. Multilevel analyses of QALYs at T1, without data imputation (n = 355). [file 12962_2019_181_MOESM4_ESM.docx]

**ADDITIONAL MATERIALS**

**Additional file 4. Multilevel analyses**

**Table S8** Multilevel analysis of well-being (SPF-ILs) at T1, after data imputation (*n* = 464)

|  | B | SE |
| --- | --- | --- |
| Constant | 2.20*** | 0.31 |
| Intervention group | -0.09** | 0.04 |
| Age | -0.01 | 0.003 |
| Sex (female) | 0.03 | 0.04 |
| Educational level (low) | -0.03 | 0.04 |
| Marital status (single) | -0.07 | 0.04 |
| Frailty score | -0.03*** | 0.01 |
| Multimorbidity | -0.02 | 0.05 |
| Well-being at T0 | 0.48*** | 0.04 |

SE, standard error; **p* < 0.05 (two-tailed); ***p* < 0.01 (two-tailed); ****p* < 0.001 (two-tailed)

Analysis shows the impact of intervention group, adjusted for age, sex, educational level, marital status, frailty score, multimorbidity, well-being at T0 and GP practice (random effect)

**Table S9** Multilevel analysis of well-being (SPF-ILs) at T1, ***without*** data imputation (*n* = 354)

|  | B | SE |
| --- | --- | --- |
| Constant | 2.28*** | 0.44 |
| Intervention group | -0.10* | 0.05 |
| Age | -0.01 | 0.01 |
| Sex (female) | 0.04 | 0.06 |
| Educational level (low) | -0.03 | 0.05 |
| Marital status (single) | -0.08 | 0.05 |
| Frailty score | -0.04*** | 0.01 |
| Multimorbidity | -0.03 | 0.08 |
| Well-being at T0 | 0.47*** | 0.05 |

SE, standard error; **p* < 0.05 (two-tailed); ***p* < 0.01 (two-tailed); ****p* < 0.001 (two-tailed)

Analysis shows the impact of intervention group, adjusted for age, sex, educational level, marital status, frailty score, multimorbidity, well-being at T0 and GP practice (random effect)

**Table S10** Multilevel analysis of QALYs at T1, after data imputation (*n* = 464)

|  | B | SE |
| --- | --- | --- |
| Constant | 1.07*** | 0.16 |
| Intervention group | -0.03 | 0.02 |
| Age | -0.01** | 0.002 |
| Sex (female) | -0.04 | 0.02 |
| Educational level (low) | 0.04* | 0.02 |
| Marital status (single) | 0.02 | 0.02 |
| Frailty score | -0.01* | 0.01 |
| Multimorbidity | -0.02 | 0.03 |
| QALYs at T0 | 0.27*** | 0.04 |

SE, standard error; **p* < 0.05 (two-tailed); ***p* < 0.01 (two-tailed); ****p* < 0.001 (two-tailed)

Analysis shows the impact of intervention group, adjusted for age, sex, educational level, marital status, frailty score, multimorbidity, QALYs at T0 and GP practice (random effect)

**Table S11** Multilevel analysis of QALYs at T1, ***without*** data imputation (*n* = 355)

|  | B | SE |
| --- | --- | --- |
| Constant | 0.95*** | 0.19 |
| Intervention group | -0.02 | 0.02 |
| Age | -0.004 | 0.002 |
| Sex (female) | -0.06* | 0.03 |
| Educational level (low) | 0.04 | 0.02 |
| Marital status (single) | 0.03 | 0.03 |
| Frailty score | -0.01* | 0.01 |
| Multimorbidity | -0.04 | 0.04 |
| QALYs at T0 | 0.35*** | 0.05 |

SE, standard error; **p* < 0.05 (two-tailed); ***p* < 0.01 (two-tailed); ****p* < 0.001 (two-tailed)

Analysis shows the impact of intervention group, adjusted for age, sex, educational level, marital status, frailty score, multimorbidity, QALYs at T0 and GP practice (random effect)
