# Supplementary material for: Cost-effectiveness of a proactive, integrated primary care approach for community-dwelling frail older persons
Source: Cost Eff Resour Alloc. 2019 Jul 9;17:14. doi: 10.1186/s12962-019-0181-8 (PMC6617694; doi:10.1186/s12962-019-0181-8)
Supplement: Supplementary file 5 — Additional file 5: Table S12. Multilevel analyses well-being, using propensity score matching (Model 1)a and data imputation (n = 463). Table S13. Multilevel analyses well-being, using propensity score matching (Model 2)a and data imputation (n = 459). Table S14. Multilevel analyses well-being, using propensity score matching (Model 1)a and without data imputation (n = 353). Table S15. Multilevel analyses well-being, using propensity score matching (Model 2)a and without data imputation (n = 349). Table S16. Multilevel analyses QALYs, using propensity score matching (Model 1)a and data imputation (n = 463). Table S17. Multilevel analyses QALYs, using propensity score matching (Model 2)a and data imputation (n = 459). Table S18. Multilevel analyses QALYs, using propensity score matching (Model 1)a and without data imputation (n = 354). Table S19. Multilevel analyses QALYs, using propensity score matching (Model 2)a and without data imputation (n = 351). [file 12962_2019_181_MOESM5_ESM.docx]

**ADDITIONAL MATERIALS**

**Additional file 5. Propensity score matching**

**Table S12** Multilevel analysis well-being, using propensity score matching (Model 1)^a^ and data imputation (*n* = 463)

|  | B | SE |
| --- | --- | --- |
| Constant | 2.19*** | 0.31 |
| Intervention group | -0.09* | 0.04 |
| Age | -0.01 | 0.004 |
| Sex (female) | 0.03 | 0.04 |
| Educational level (low) | -0.03 | 0.04 |
| Marital status (single) | -0.07 | 0.04 |
| Frailty score | -0.03*** | 0.01 |
| Multimorbidity | -0.02 | 0.05 |
| Well-being at T0 (SPF-ILs) | 0.48*** | 0.04 |

SE, standard error; **p* < 0.05 (two-tailed); ***p* < 0.01 (two-tailed); ****p* < 0.001 (two-tailed)

^a^Logistic regression model 1 with covariates: age, sex, marital status, educational level, frailty score, and multimorbidity

**Table S13** Multilevel analysis well-being, using propensity score matching (Model 2)^a^ and data imputation (*n* = 459)

|  | B | SE |
| --- | --- | --- |
| Constant | 2.16*** | 0.32 |
| Intervention group | -0.09* | 0.04 |
| Age | -0.01 | 0.004 |
| Sex (female) | 0.04 | 0.04 |
| Educational level (low) | -0.03 | 0.04 |
| Marital status (single) | -0.08 | 0.04 |
| Frailty score | -0.03*** | 0.01 |
| Multimorbidity | -0.02 | 0.05 |
| Well-being at T0 (SPF-ILs) | 0.49*** | 0.04 |

SE, standard error; **p* < 0.05 (two-tailed); ***p* < 0.01 (two-tailed); ****p* < 0.001 (two-tailed)

^a^Logistic regression model 2 with covariates: age, sex, marital status, educational level, frailty score, and multimorbidity, and baseline SPF-ILs, QALYs and costs

**Table S14** Multilevel analysis well-being, using propensity score matching (Model 1)^a^ and ***without*** data imputation (*n* = 353)

|  | B | SE |
| --- | --- | --- |
| Constant | 2.27*** | 0.44 |
| Intervention group | -0.10* | 0.05 |
| Age | -0.01 | 0.01 |
| Sex (female) | 0.04 | 0.06 |
| Educational level (low) | -0.03 | 0.05 |
| Marital status (single) | -0.08 | 0.05 |
| Frailty score | -0.04*** | 0.01 |
| Multimorbidity | -0.03 | 0.08 |
| Well-being at T0 (SPF-ILs) | 0.47*** | 0.05 |

SE, standard error; **p* < 0.05 (two-tailed); ***p* < 0.01 (two-tailed); ****p* < 0.001 (two-tailed)

^a^Logistic regression model 1 with covariates: age, sex, marital status, educational level, frailty score, and multimorbidity

**Table S15** Multilevel analysis well-being, using propensity score matching (Model 2)^a^ and ***without*** data imputation (*n* = 349)

|  | B | SE |
| --- | --- | --- |
| Constant | 2.22*** | 0.44 |
| Intervention group | -0.10* | 0.05 |
| Age | -0.01 | 0.01 |
| Sex (female) | 0.05 | 0.06 |
| Educational level (low) | -0.03 | 0.05 |
| Marital status (single) | -0.09 | 0.05 |
| Frailty score | -0.04*** | 0.01 |
| Multimorbidity | -0.03 | 0.08 |
| Well-being at T0 (SPF-ILs) | 0.49*** | 0.05 |

SE, standard error; **p* < 0.05 (two-tailed); ***p* < 0.01 (two-tailed); ****p* < 0.001 (two-tailed)

^a^Logistic regression model 2 with covariates: age, sex, marital status, educational level, frailty score, and multimorbidity, and baseline SPF-ILs, QALYs and costs

**Table S16** Multilevel analysis QALYs, using propensity score matching (Model 1)^a^ and data imputation (*n* = 463)

|  | B | SE |
| --- | --- | --- |
| Constant | 1.07*** | 0.16 |
| Intervention group | -0.03 | 0.02 |
| Age | -0.01** | 0.002 |
| Sex (female) | -0.04 | 0.02 |
| Educational level (low) | 0.04* | 0.02 |
| Marital status (single) | 0.02 | 0.02 |
| Frailty score | -0.01* | 0.01 |
| Multimorbidity | -0.02 | 0.03 |
| QALYs at T0 (utilities based on EQ-5D-3L) | 0.26*** | 0.04 |

SE, standard error; **p* < 0.05 (two-tailed); ***p* < 0.01 (two-tailed); ****p* < 0.001 (two-tailed)

^a^Logistic regression model 1 with covariates: age, sex, marital status, educational level, frailty score, and multimorbidity

**Table S17** Multilevel analysis QALYs, using propensity score matching (Model 2)^a^ and data imputation (*n* = 459)

|  | B | SE |
| --- | --- | --- |
| Constant | 1.05*** | 0.16 |
| Intervention group | -0.03 | 0.02 |
| Age | -0.01** | 0.002 |
| Sex (female) | -0.05* | 0.02 |
| Educational level (low) | 0.04* | 0.02 |
| Marital status (single) | 0.02 | 0.02 |
| Frailty score | -0.01* | 0.004 |
| Multimorbidity | -0.02 | 0.03 |
| QALYs at T0 (utilities based on EQ-5D-3L) | 0.25*** | 0.04 |

SE, standard error; **p* < 0.05 (two-tailed); ***p* < 0.01 (two-tailed); ****p* < 0.001 (two-tailed)

^a^Logistic regression model 2 with covariates: age, sex, marital status, educational level, frailty score, and multimorbidity, and baseline SPF-ILs, QALYs and costs

**Table S18** Multilevel analysis QALYs, using propensity score matching (Model 1)^a^ and ***without*** data imputation (*n* = 354)

|  | B | SE |
| --- | --- | --- |
| Constant | 0.95*** | 0.19 |
| Intervention group | -0.02 | 0.02 |
| Age | -0.004 | 0.002 |
| Sex (female) | -0.06* | 0.03 |
| Educational level (low) | 0.04 | 0.02 |
| Marital status (single) | 0.03 | 0.03 |
| Frailty score | -0.01* | 0.01 |
| Multimorbidity | -0.04 | 0.04 |
| QALYs at T0 (utilities based on EQ-5D-3L) | 0.35*** | 0.05 |

SE, standard error; **p* < 0.05 (two-tailed); ***p* < 0.01 (two-tailed); ****p* < 0.001 (two-tailed)

^a^Logistic regression model 1 with covariates: age, sex, marital status, educational level, frailty score, and multimorbidity

**Table S19** Multilevel analysis QALYs, using propensity score matching (Model 2)^a^ and ***without*** data imputation (*n* = 351)

|  | B | SE |
| --- | --- | --- |
| Constant | 0.91*** | 0.19 |
| Intervention group | -0.02 | 0.02 |
| Age | -0.004 | 0.002 |
| Sex (female) | -0.06* | 0.03 |
| Educational level (low) | 0.04 | 0.02 |
| Marital status (single) | 0.03 | 0.03 |
| Frailty score | -0.009 | 0.01 |
| Multimorbidity | -0.04 | 0.04 |
| QALYs at T0 (utilities based on EQ-5D-3L) | 0.34*** | 0.05 |

SE, standard error; **p* < 0.05 (two-tailed); ***p* < 0.01 (two-tailed); ****p* < 0.001 (two-tailed)

^a^Logistic regression model 2 with covariates: age, sex, marital status, educational level, frailty score, and multimorbidity, and baseline SPF-ILs, QALYs and costs
