# Supplementary material for: Cost-effectiveness of a proactive, integrated primary care approach for community-dwelling frail older persons
Source: Cost Eff Resour Alloc. 2019 Jul 9;17:14. doi: 10.1186/s12962-019-0181-8 (PMC6617694; doi:10.1186/s12962-019-0181-8)
Supplement: Supplementary file 6 — Additional file 6: Table S20. Analyses of participants of intervention GP practices with high degree of implementation and matched controls without data imputation. Table S21. Healthcare costs (in euros) of participants of intervention GP practices with high degree of implementation and matched controls without data imputation. Table S22. Analyses of participants of intervention GP practices with high degree of implementation and matched controls, after data imputation. Table S23. Healthcare costs (in euros) of participants of intervention GP practices with high degree of implementation and matched controls, after data imputation. [file 12962_2019_181_MOESM6_ESM.docx]

**ADDITIONAL MATERIALS**

**Additional file 6. Analyses based on subgroup of intervention GP practices with high degree of implementation**

**Table S20** Analyses of participants of intervention GP practices with high degree of implementation and matched controls ***without*** data imputation

|  |  | **Care as usual** | ***n*** | **FFF approach** | ***n*** |
| --- | --- | --- | --- | --- | --- |
| **Outcome measures** |  |  |  |  |  |
| Well-being (SPF-ILs) | T0 | 2.62 (0.45) | 92 | 2.65 (0.48) | 91 |
|  | T1 | 2.70 (0.53) | 68 | 2.70 (0.53) | 79 |
| QALYs  (utilities based on EQ-5D-3L) | T0 | 0.66 (0.24) | 92 | 0.64 (0.28) | 93 |
|  | T1 | 0.70 (0.23) | 68 | 0.70 (0.25) | 80 |

Values are presented as mean (SD)

SPF-ILs: Social Production Function Instrument for the Level of well-being short (range, 1-4); EQ-5D-3L: five-dimensional three-level EuroQol (range for utilities, -0.33-1)

**p* < 0.05 (two-tailed)

Independent samples *t*-tests

**Table S21** Healthcare costs (in euros) of participants of intervention GP practices with high degree of implementation and matched controls ***without*** data imputation

|  | **Care as usual** | ***n*** | **FFF approach** | ***n*** |
| --- | --- | --- | --- | --- |
| **Healthcare costs** |  |  |  |  |
| Mean total costs at T0^a^ | 8085.43 (12,226.46) | 70 | 7736.96 (10,915.11) | 80 |
| Mean total costs at T1^a^ | 8989.07 (12,264.61) | 65 | 9942.37 (14,985.58) | 76 |

^a^Means (SDs) were calculated including persons without healthcare utilization

**Table S22** Analyses of participants of intervention GP practices with high degree of implementation and matched controls, after data imputation

|  |  | **Care as usual** | ***n*** | **FFF approach** | ***n*** |
| --- | --- | --- | --- | --- | --- |
| **Outcome measures** |  |  |  |  |  |
| Well-being (SPF-ILs) | T0 | 2.62 (0.45) | 93 | 2.65 (0.48) | 93 |
|  | T1 | 2.67 (0.48) | 93 | 2.67 (0.51) | 93 |
| QALYs  (utilities based on EQ-5D-3L) | T0 | 0.66 (0.23) | 93 | 0.64 (0.28) | 93 |
|  | T1 | 0.70 (0.21) | 93 | 0.68 (0.25) | 93 |

Values are presented as mean (SD)

SPF-ILs: Social Production Function Instrument for the Level of well-being short (range, 1-4); EQ-5D-3L: five-dimensional three-level EuroQol (range for utilities, -0.33-1)

**p* < 0.05 (two-tailed)

Independent samples *t*-tests

**Table S23** Healthcare costs (in euros) of participants of intervention GP practices with high degree of implementation and matched controls, after data imputation

|  | **Care as usual** | ***n*** | **FFF approach** | ***n*** |
| --- | --- | --- | --- | --- |
| **Healthcare costs** |  |  |  |  |
| Mean total costs at T0^a^ | 8940.79 (11,902.79) | 93 | 8804.80 (12,128.38) | 93 |
| Mean total costs at T1^a^ | 10,337.77 (12,419.17) | 93 | 10,060.00 (13,858.17) | 93 |

^a^Means (SDs) were calculated including persons without healthcare utilization
